# Southern Dental Journal

**Published:** 1890-04

**Authors:** 


### SOUTHERN DENTAL JOURNAL.

Dr. Catching, editor of the *Southern Dental Journal* since its inception, has resigned in favor of Dr. H. H. Johnson, who, in assuming the editorial position, says, in answer to a query from the *Archives*,—

"It is true there has been a change in the editorial department, but its present management is determined that it shall not fall below its former high standard of excellence thereby, and by diligence and care we hope to see it improve as it grows in age, and to keep apace with the profession as it advances to a higher standard of perfection."

Dr. Catching does not intend to retire from journalistic work entirely, but will issue an annual review of dentistry in the form of a compendium which will be sold on subscription only. There seems to be a field for such a work, and Dr. Catching will undoubtedly make it a success. We wish both the fullest success in their new fields.

